# 超高效液相色谱-四极杆-飞行时间质谱法快速筛查与定量分析烟叶中26种可溶性糖

**DOI:** 10.3724/SP.J.1123.2025.10022

**Published:** 2026-05-08

**Authors:** Pengfei YANG, Zhengbo FENG, Cheng FANG, Yake LUO, Jiaxiang FEI, Duobin MAO

**Affiliations:** 1.郑州轻工业大学烟草科学与工程学院，河南 郑州，450001; 1. College of Tobacco Science and Engineering，Zhengzhou University of Light Industry，Zhengzhou 450001，China; 2.深圳烟草工业有限责任公司技术研发中心，广东 深圳，518000; 2. Technology R＆D Center，Shenzhen Tobacco Industrial Co. ，Ltd. ，Shenzhen 518000，China

**Keywords:** 超高效液相色谱-四极杆-飞行时间质谱, 可溶性糖, 快速筛查, 烟叶, 定量检测, ultra performance liquid chromatography-quadrupole-time of flight mass spectrometry （UPLC-Q-TOF-MS）, soluble sugars, rapid screen, tobacco leaves, quantitative detection

## Abstract

烟草中糖类物质的组成及含量对于烟草品质至关重要。本研究基于超高效液相色谱-四极杆-飞行时间质谱（UPLC-Q-TOF-MS）技术结合自建数据库建立了同时快速筛查与准确定量烟叶中26种可溶性糖的分析方法。烟叶样品采用30 mL 40%乙腈水溶液，在120 W提取功率下超声提取30 min，用Waters Sep-Pak C18固相萃取小柱处理得到可溶性糖提取物。提取物经UPLC-Q-TOF-MS分析，结合自建数据库对烟叶样品进行筛查，并以基质匹配标准曲线进行定量。结果表明，26种可溶性糖具有良好的线性关系，相关系数（*R* ^2^）为0.999 1~0.999 9，定量限为0.03~20 mg/L，加标回收率为92.39%~111.75%，相对标准偏差≤4.65%，该方法展示出优异的准确度与精密度。将该方法用于不同产地、等级及年份的59种烟叶样品中，共筛查并获得6种可溶性糖的含量。定量结果表明，各可溶性糖含量在不同产地、等级和年份间存在显著差异，即使同一产地、等级和年份的不同样本间，可溶性糖含量也存在一定波动。其中果糖和葡萄糖在所有烟叶样品中含量均较高；黑龙江地区烟叶可溶性糖含量普遍高于贵州、湖南等地区；2021年和2022年烟叶可溶性糖含量整体高于2020年。选取不同部位烟叶样品进行主成分分析与层次聚类分析，结果表明不同烟叶部位中可溶性糖含量差异显著，证明可通过可溶性糖区分烟叶不同部位。该方法具有高通量、简单、快捷、准确的特点，适用于烟叶样品的可溶性糖类定性定量检测，可为烟草化学成分的深入研究与卷烟数字化设计提供有力的技术支撑。

## Abstract (en)

Sugars are one of the important chemical components in tobacco. They serve as key precursor substances for various aromatic compounds in tobacco. Furthermore， they also have a significant impact on the taste and burning characteristics of tobacco. Thus， obtaining accurate information on the composition and content of soluble sugars in tobacco can provide a scientific basis for quality assessment of tobacco leaves. Moreover， it offers critical data support for quality control and process optimization. Traditional techniques for soluble sugar analysis include continuous flow analysis， ion chromatography， gas chromatography， and liquid chromatography， etc. However， these methods still have limitations such as tedious sample pretreatment， poor detection sensitivity， and low analysis efficiency. In contrast， ultra performance liquid chromatography-quadrupole-time of flight mass spectrometry （UPLC-Q-TOF-MS） is a highly effective analytical technology. It integrates the excellent separation capability of UPLC and the superior sensitivity of MS. This combination offers great potential for qualitative and quantitative analysis of target compounds. Nonetheless， the types of soluble sugars detectable in tobacco by UPLC-Q-TOF-MS remain limited. This defect restricts the comprehensive evaluation of sugar components in tobacco. Herein， a rapid and accurate analysis method was established for high-throughput screening and quantification of 26 soluble sugars in tobacco leaves. The tobacco leaf samples were ultrasonically extracted with 30 mL 40% acetonitrile aqueous solution at 120 W for 30 min. Soluble sugar extract solution was prepared using a Waters Sep-Pak C18 solid-phase extraction cartridge. With the help of self-built sugar library， the tobacco leaf samples were screened by UPLC-Q-TOF-MS. Then matrix-matched standard calibration curves were employed to accurately quantify the target analytes. The established method was verified. The 26 soluble sugars exhibited good linear relationships with correlation coefficients （*R* ^2^） ranging from 0.999 1 to 0.999 9. The limits of quantification （LOQs） were in the range of 0.03-20 mg/L. The spiked recoveries ranged from 92.39% to 111.75%， and relative standard deviations （RSDs） were ≤4.65%. Finally， this method was applied to analyze 59 tobacco leaf samples from different origins， grades and years. Six soluble sugars including erythritol， fructose， sorbitol， glucose， sucrose and inositol were successfully identified. The content of each soluble sugar varied significantly among samples from different origins， grades， and years. Even among samples from the same origin， grade， and year， the soluble sugar contents showed certain fluctuations. This is speculated to be related to factors such as the growth environment， maturity， processing， and harvest time of the tobacco leaves. Among them， the contents of fructose and glucose were at relatively high levels in all tobacco leaf samples. The soluble sugar content from Heilongjiang was generally higher than that from Guizhou， Hunan， and other regions. Additionally， the overall soluble sugar content in samples from 2021 and 2022 was higher than that from 2020. Principal component analysis （PCA） and hierarchical cluster analysis （HCA） were conducted on tobacco leaf samples from different positions， respectively. These analyses demonstrated significant differences in soluble sugar contents among tobacco leaves from different positions. It confirmed that soluble sugars could serve as effective indicators for distinguishing positions of tobacco leaves. In summary， the established method enables the qualitative and quantitative detection of the 26 soluble sugars without relying on standard substances. Moreover， the method exhibits high throughput， simplicity， rapidity， and accuracy. Meanwhile， it is suitable for the qualitative and quantitative detection of soluble sugars in tobacco leaf samples. It provides robust technical support for in-depth research on tobacco chemical components and the digital formulation design of cigarette products.

糖类物质是烟草中含量最丰富的化学成分，约占调制后烟叶的25%~50%，主要以葡萄糖、果糖、麦芽糖等可溶性糖存在^［1］^。作为烟草生长发育和卷烟品质形成的关键物质，糖类物质在烟草中发挥着重要作用^［2］^。在烟草生长过程中，糖类物质的组成和含量会随着品种、生长阶段、烟叶部位以及环境条件的不同而发生变化，可溶性糖含量变化可以反映烟叶的成熟度和品质。在卷烟加工及燃吸阶段，糖类物质的作用尤为重要^［3］^。在卷烟加工过程中，糖类物质具有增香保润及增加烟叶弹性的作用，从而影响卷烟品质^［4］^。糖类物质作为多种致香成分的重要前体物，在燃烧过程中会通过美拉德反应产生多种风味化合物，其组成和含量对卷烟的风格和口感具有重要作用^［5］^。化学分析表明，烟叶商品等级与含糖量呈正相关^［6］^。因此，准确了解烟草中可溶性糖组成和含量，不仅可为烟叶质量评价提供科学依据，还能为品质调控、工艺优化等提供重要参考，对提升烟草产业技术水平具有重要实践意义。

烟草行业一般采用连续流动分析仪测定可溶性总糖和还原糖的百分含量，但该方法无法区分及准确定量每种糖含量^［7，8］^。传统的测定可溶性糖的技术有离子色谱^［9，10］^、气相色谱^［11］^、液相色谱-蒸发光散射检测器（HPLC-ELSD）^［12-14］^等，但仍存在样品前处理烦琐、检测灵敏度不足、分析效率低等局限性。超高效液相色谱-质谱联用技术（UPLC-MS）是一种将超高效液相色谱强大的分离能力和质谱优异的灵敏度集为一体的方法，在分析物定性和定量能力方面展现出巨大的潜力^［15］^。曾婉俐等^［16］^建立了超高效液相色谱-串联质谱法，成功用于云产卷烟样品中果糖、葡萄糖、蔗糖、麦芽糖4种水溶性糖的含量测定。赵瑜等^［17］^建立了一种能够同时快速测定烟叶及卷烟中7种主要糖类物质的液相色谱-串联质谱的定量分析方法。然而，目前基于液相色谱-串联质谱技术的烟草糖类分析方法检测的可溶性糖种类有限，制约了该技术在烟草糖类成分综合评价中的深入应用，因此亟须开发更全面、更灵敏的糖类分析新方法以满足烟草品质研究需求。

本研究在已构建可溶性糖筛查数据库的基础上^［18］^，基于超高效液相色谱-四极杆-飞行时间质谱（UPLC-Q-TOF-MS）技术进一步建立了26种可溶性糖的定量分析方法。通过对提取溶剂、提取溶剂体积、提取时间和提取功率、固相萃取小柱种类等影响可溶性糖提取效率的参数考察，确定最优提取条件。最终，将该方法用于烟叶不同部位中可溶性糖的检测。建立的定量检测方法在数据库辅助快速定性的基础上，兼具操作简单、分析高效和结果准确的优势，为筛选烟叶原料质量、评估烟草品质提供了强有力的工具。

## 1 实验部分

### 1.1 仪器、试剂与材料

1290 Infinity Ⅱ超高效液相色谱（美国Agilent公司）；Triple TOF 6600^+^ MS（美国SCIEX公司）；KQ-5200DB型超声波清洗仪（中国昆山市超声仪器有限公司）；台式高速离心机（德国Eppendorf公司）；PTX-FA210S型分析天平（瑞士Mettler Toledo公司）；Milli-Q超纯水系统（美国Millipore公司）；Waters Sep-Pak C18（500 mg/6 mL，美国Waters公司）。

可溶性糖类标准品：葡萄糖（glucose）、果糖（fructose）（纯度>98%，北京百灵威科技有限公司）；半乳糖（galactose）、山梨糖（sorbose）、甘露糖（mannose）、木糖（xylose）、阿拉伯糖（arabinose）、来苏糖（lyxose）、核糖（ribose）、2-脱氧核糖（2-deoxy-dribose）、鼠李糖（rhamnose）、麦芽糖（maltose）、蔗糖（sucrose）、乳糖（lactose）、纤维二糖（cellobiose）、海藻糖（trehalose）、蜜二糖（melibiose）、肌醇（inositol）、山梨糖醇（sorbitol）、木糖醇（xylitol）、阿拉伯糖醇（arabinitol）、赤藓糖醇（erythritol）、棉子糖（raffinose）、麦芽三糖（maltotriose）、麦芽四糖（maltotetraose）、水苏糖（stachyose）（纯度>98%，上海麦克林生化科技有限公司）；乙腈（色谱级，美国Fisher公司）；氨水（色谱级，北京百灵威科技有限公司）。

所用烟叶样品均由广东中烟工业有限责任公司提供，产地如下：No. 1美国，No. 2巴西，No. 3~7津巴布韦，No. 8~31中国云南，No. 32~39中国四川，No. 40~41中国广东，No. 42~45中国贵州，No. 46~49中国福建，No. 50~55中国湖南，No. 56~57中国黑龙江，No. 58中国陕西，No. 59中国山东。

### 1.2 实验方法

#### 1.2.1 标准溶液制备

准确称取26种可溶性糖标准品各5 mg于10 mL棕色容量瓶中，以75%乙腈水溶液为溶剂，配制成质量浓度为0.5 mg/mL的标准储备溶液，于-18 ℃冰箱中储存备用。

取与待测样品相同条件前处理后的烟叶样品进行Waters Sep-Pak C18固相萃取小柱处理，收集组分作为对照基质溶液。分别取一定量的各可溶性糖标准储备溶液，用对照基质溶液逐级稀释成系列标准溶液。

#### 1.2.2 样品前处理

取烟叶样品，在30 ℃下烘干后粉碎并过60目筛。移取30 mL 40%乙腈水溶液置于含有1 g烟末样品的锥形瓶中，超声提取30 min，提取功率为120 W，经Waters Sep-Pak C18固相萃取小柱富集纯化后得到可溶性糖提取物。将获得的提取物用对照基质溶液梯度稀释后过0.22 µm有机系滤膜，待UPLC-MS/MS分析，平行3次（对于含量不同的分析物测定时稀释倍数有所差异）。

#### 1.2.3 分析条件

XBridge Amide柱（150 mm×4.6 mm， 3.5 μm，美国Waters公司）；流动相：0.15%氨水溶液（流动相A）和乙腈溶液（流动相B）；流速：0.8 mL/min；进样量：3.0 μL；柱温：45 ℃；运行时间：40 min；梯度洗脱程序：0~20 min，90%B~75%B；20~25 min，75%B~70%B；25~35 min，70%B~60%B；35~37 min，60%B~90%B；37~40 min，90%B。

离子源：电喷雾；电离模式：ESI^+^；离子源温度：600 ℃；扫描范围：*m/z* 50~750；喷雾气压力：482 kPa；辅助加热气压力：482 kPa；气帘气压力：276 kPa；离子化电压：5 500 V，去簇电压（MS^1^）：40 V，碰撞能量（MS^1^）：5 V，去簇电压（MS^2^）：40 V，碰撞能量（MS^2^）：（12±5） V；固定采集窗口：14个。

### 1.3 数据处理

由Analyst^TM^ TF软件采集实验数据，通过带有XIC Manager模块的Peak View（Version 2.6 AB SCIEX）筛查目标物，利用MultiQuant（Version 3.0.2 AB SCIEX）软件对筛查出的目标化合物进行定量分析，采用GraphPad Prism和Origin软件进行绘图。使用Simca软件进行主成分分析（PCA）、层次聚类分析（HCA）。

## 2 结果与讨论

### 2.1 样品前处理优化

#### 2.1.1 提取条件优化

影响提取效率的主要因素包括提取溶剂、提取溶剂体积、提取时间和提取功率等。鉴于醛糖和酮糖是后期烟叶醇化过程中参与美拉德反应的反应物，本研究选取葡萄糖和果糖分别作为醛糖和酮糖的代表性成分，以其色谱峰面积为评价指标，对上述4个因素进行考察。通过单因素实验初步获得各因素的有效范围，并进一步采用正交实验设计对上述4个因素进行系统优化，以确定最佳提取条件。

本研究首先对提取溶剂（0~100%的乙腈水溶液）进行考察。由图1可以看出，随着乙腈比例的增加，烟叶中葡萄糖和果糖的峰面积均呈现先升高后降低的趋势。当用50%乙腈水溶液和75%的乙腈水溶液进行提取时，葡萄糖和果糖的峰面积几乎相当。因此，拟选定50%乙腈水溶液作为提取溶剂。

**图1:**
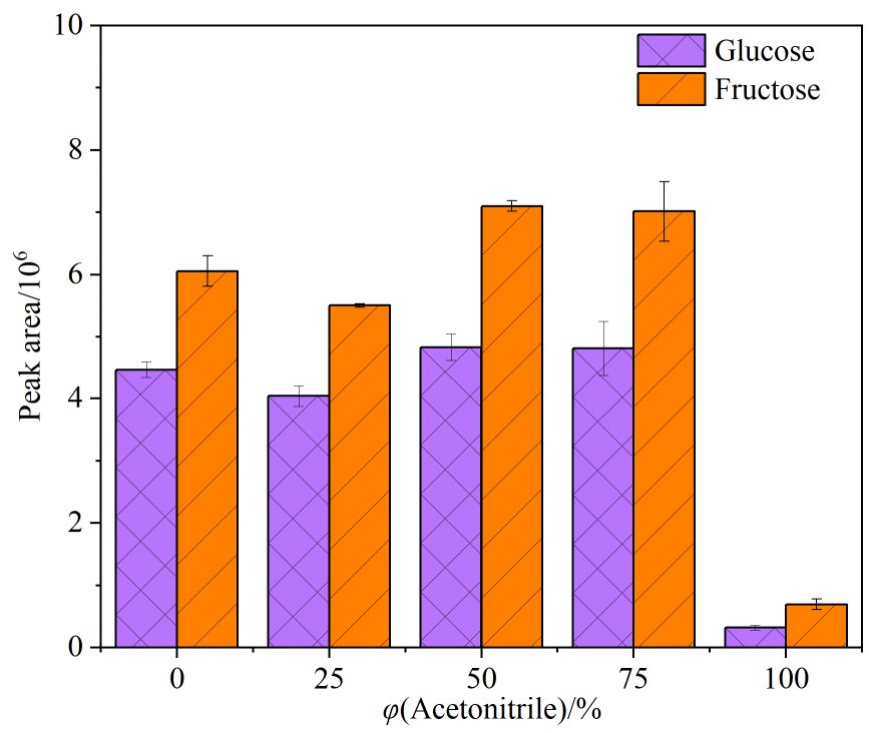
提取溶剂对葡萄糖和果糖峰面积的影响（*n*=3）

提取溶剂体积的优化结果如图2所示。从图中可以看出，随着提取溶剂体积的增加，葡萄糖和果糖的峰面积呈现先升高后降低的趋势。当提取溶剂体积为40 mL时，葡萄糖和果糖峰面积最高。这可能是由于在较低的提取溶剂体积下，溶剂不足以充分溶解样品中的葡萄糖和果糖，而随着提取溶剂体积的增加，样品的溶解量提高，峰面积相应增加。然而，当提取溶剂体积过高时，溶剂量过大可能会加剧其他物质溶出，导致葡萄糖和果糖峰面积下降。因此，提取溶剂体积设为40 mL。

**图2:**
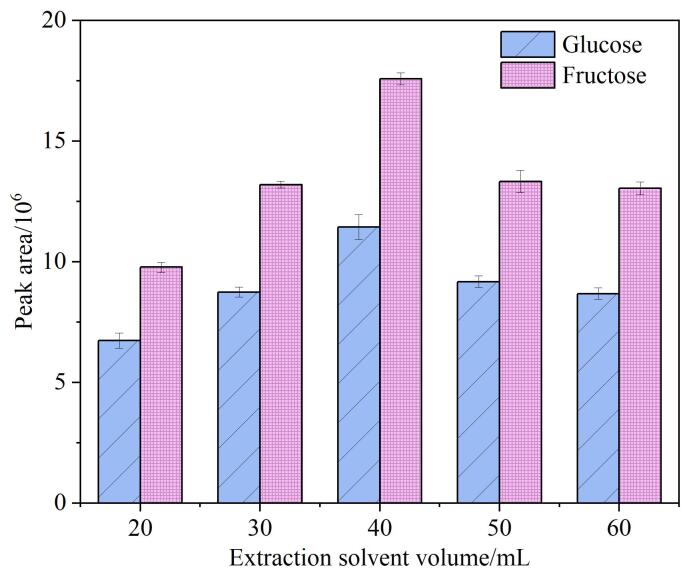
提取溶剂体积对葡萄糖和果糖峰面积的影响（*n*=3）

提取时间的优化结果如图3所示。从图中可以看出，随着提取时间的增加，葡萄糖和果糖的峰面积呈现先升高后下降的趋势，当提取时间为30 min时峰面积达到最大值。因此，提取时间设为30 min。

**图3:**
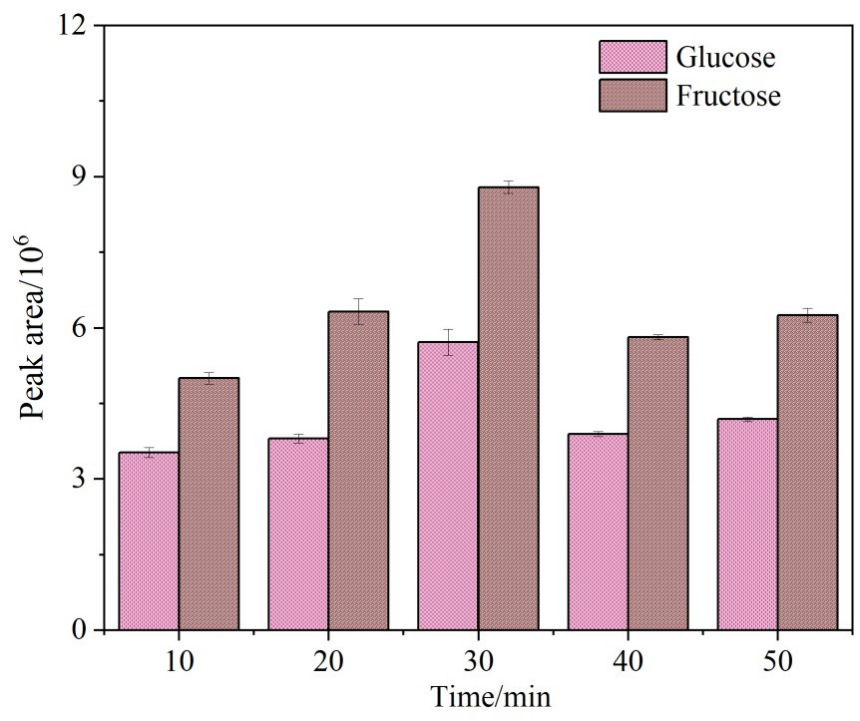
提取时间对葡萄糖和果糖峰面积的影响（*n*=3）

提取功率的优化结果如图4所示。从图中可以看出，随着提取功率的增大，葡萄糖和果糖的峰面积呈现先升高后下降的趋势，当超声功率为100 W时，葡萄糖和果糖的峰面积达到最高。因此，提取功率设为100 W。

**图4:**
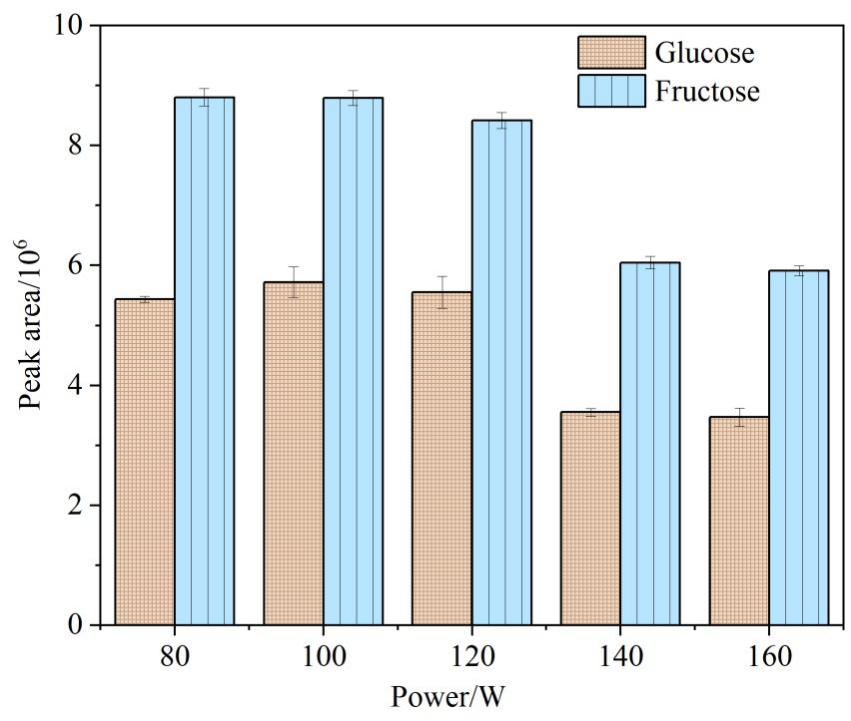
提取功率对葡萄糖和果糖峰面积的影响（*n*=3）

综合单因素实验结果，设计四因素三水平L_9_（3^4^）正交实验考察提取溶剂、提取溶剂体积、提取时间、提取功率对葡萄糖和果糖提取效率的影响（表1）。由极差*R*的大小可知，各因素对烟叶中葡萄糖和果糖提取效率的影响顺序如下：提取功率>提取溶剂体积>提取时间>提取溶剂，即提取功率是影响提取效率最主要的因素，提取溶剂影响最小。4个因素的最优提取组合为40%乙腈水溶液，提取溶剂体积30 mL，提取时间30 min，提取功率120 W。因此，最终确定该条件为烟叶中可溶性糖的提取条件。

**表 1:** 正交实验结果（*n*=3）

No.	Extraction solvent/%	Extraction solvent volume/mL	Extraction time/min	Extraction power/W	Peak areas	
					Glucose	Fructose
1	40	30	20	80	5.9×10^6^	8.6×10^6^
2	40	40	30	100	7.1×10^6^	1.0×10^7^
3	40	50	40	120	7.0×10^6^	1.0×10^7^
4	50	30	30	120	7.5×10^6^	1.1×10^7^
5	50	40	40	80	5.5×10^6^	8.1×10^6^
6	50	50	20	100	6.5×10^6^	9.4×10^6^
7	60	30	40	100	6.9×10^6^	9.9×10^6^
8	60	40	20	120	7.4×10^6^	1.0×10^7^
9	60	50	30	80	5.6×10^6^	8.3×10^6^
*K*_1_	1.62×10^7^	1.68×10^7^	1.59×10^7^	1.40×10^7^		
*K*_2_	1.60×10^7^	1.61×10^7^	1.65×10^7^	1.66×10^7^		
*K*_3_	1.60×10^7^	1.56×10^7^	1.58×10^7^	1.73×10^7^		
*R*	2.0×10^5^	1.2×10^6^	7.0×10^5^	3.3×10^6^		

#### 2.1.2 固相萃取小柱选择

为了降低样品基质对目标物检测的干扰，分别选取Agilent Bond Elut C18 （1 g/6 mL）、Agilent Bond Elut SI （1 g/6 mL）、Agilent Bond Elut NH_2_ （1 g/6 mL）、Waters Sep-Pak C18 （500 mg/6 mL）、Waters Oasis HLB （500 mg/6 mL）5种固相萃取小柱进行考察，以经固相萃取处理的样品为实验组，以未经固相萃取处理但经相同提取、稀释及定容步骤所得样品为对照组，计算二组葡萄糖和果糖的相对峰面积比，以此作为固相萃取效果的评价指标。

不同固相萃取小柱的净化结果如图5所示。从图中可以看出，Agilent Bond Elut SI和Waters Oasis HLB小柱对葡萄糖和果糖的回收率相对较低，而Agilent Bond Elut C18、Waters Sep-Pak C18和Agilent Bond Elut NH_2_小柱对葡萄糖和果糖的回收率均高于90%，其中经Waters Sep-Pak C18小柱净化的回收率最高。这归因于Waters Sep-Pak C18小柱对非极性化合物有较强的保留能力，可以有效吸附样品中的非极性杂质，而对极性较强的糖类物质保留较差，从而减少目标物损失，提高回收率。因此，最终选择Waters Sep-Pak C18固相萃取小柱用于后续样品处理。

**图5:**
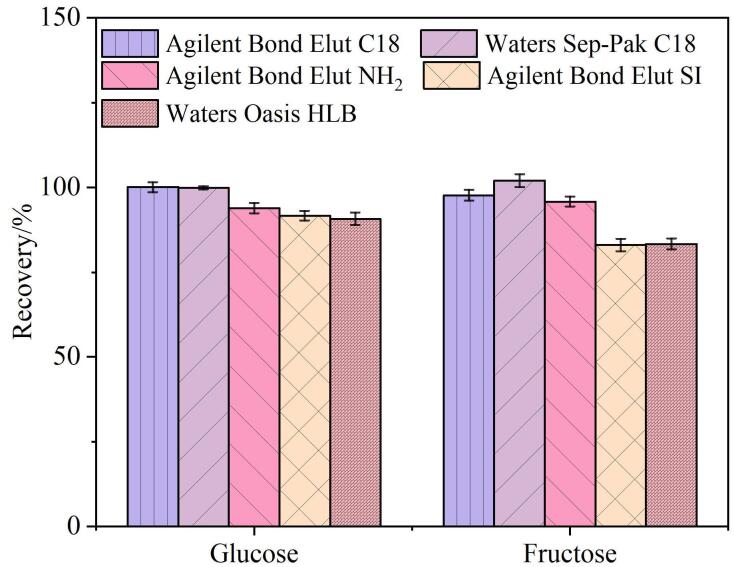
固相萃取小柱对葡萄糖和果糖回收率的影响（*n*=3）

### 2.2 方法学考察

烟叶样品中可溶性糖的共流出物会与可溶性糖类成分竞争电荷，影响可溶性糖类成分的离子化效率，从而造成定量不准确。课题组前述实验证实正离子模式下有16种糖为弱基质效应，9种糖为中等基质效应，1种糖为强基质效应^［18］^。为减小基质效应对目标物定量的影响，最终采用基质匹配标准曲线校准目标化合物含量。以各可溶性糖的质量浓度为横坐标、色谱峰面积（扣除对照基质溶液峰面积后）为纵坐标绘制标准曲线。如表2所示，26种目标物线性关系良好，相关系数（*R* ^2^）均≥0.999 1。对基质匹配标准溶液进行分析，以26种可溶性糖色谱峰面积的信噪比为3计算检出限、信噪比为10计算定量限。结果表明，26种可溶性糖的检出限为0.01~6.00 mg/L，定量限为0.03~20.0 mg/L。

**表 2:** 26种可溶性糖在烟叶基质中的线性范围、线性方程、相关系数、检出限、定量限、回收率和相对标准偏差（*n*=3）

Compound	Linear range/（mg/L）	Linear equation	*R* ^2^	LOD/（mg/L）	LOQ/（mg/L）	Recovery/%	RSD/%
2-Deoxy-dribose	25-300	*Y*=2.29×10^4^ *X*+1.60×10^5^	0.9999	3.00	10.0	100.31	1.07
Ribose	5-50	*Y*=4.19×10^4^ *X*-1.09×10^4^	0.9998	1.50	5.00	100.60	2.12
Rhamnose	30-250	*Y*=1.10×10^4^ *X*+3.50×10^4^	0.9999	6.00	20.0	100.59	1.40
Erythritol	1-10	*Y*=4.84×10^5^ *X*+3.95×10^4^	0.9994	0.10	0.30	98.62	4.65
Lyxose	25-150	*Y*=6.97×10^3^ *X*+3.01×10^4^	0.9993	3.00	10.0	100.93	4.07
Xylose	25-300	*Y*=1.05×10^4^ *X*+8.06×10^4^	0.9997	3.00	10.0	101.99	1.64
Arabinose	20-200	*Y*=2.18×10^4^ *X*-1.58×10^3^	0.9999	3.00	10.0	101.79	1.77
Xylitol	25-300	*Y*=1.63×10^4^ *X*+1.54×10^5^	0.9993	0.75	2.50	100.08	1.33
Arabinitol	25-300	*Y*=7.08×10^3^ *X*+7.64×10^4^	0.9997	0.75	2.50	100.12	0.93
Fructose	30-250	*Y*=5.38×10^4^ *X*+3.73×10^5^	0.9991	0.30	1.00	103.54	4.46
Sorbose	10-120	*Y*=2.81×10^4^ *X*+2.61×10^4^	0.9997	0.30	1.00	98.85	3.48
Mannose	0.5-10	*Y*=3.20×10^5^ *X*-3.60×10^4^	0.9998	0.30	1.00	111.75	1.78
Sorbitol	0.3-4	*Y*=3.32×10^7^ *X*+1.36×10^6^	0.9993	0.01	0.03	103.22	3.41
Glucose	20-200	*Y*=5.67×10^4^ *X*+2.79×10^5^	0.9998	1.00	3.00	105.15	3.81
Galactose	25-300	*Y*=1.39×10^5^ *X*+8.77×10^5^	0.9998	0.06	2.00	99.72	0.87
Sucrose	2-14	*Y*=6.91×10^5^ *X*+1.09×10^5^	0.9999	0.30	1.00	101.92	3.40
Inositol	1-20	*Y*=6.56×10^4^ *X*-1.04×10^4^	0.9999	0.06	2.00	102.39	4.34
Maltose	5-50	*Y*=9.17×10^5^ *X*+2.39×10^6^	0.9994	0.12	4.00	92.39	1.92
Cellobiose	10-100	*Y*=8.64×10^5^ *X*+1.67×10^6^	0.9993	3.00	10.0	99.98	1.20
Lactose	10-120	*Y*=9.76×10^4^ *X*+2.12×10^5^	0.9994	0.75	2.50	106.43	3.75
Trehalose	0.5-10	*Y*=1.28×10^6^ *X*+2.99×10^3^	0.9999	0.30	1.00	107.90	3.06
Melibiose	0.5-10	*Y*=1.36×10^6^ *X*-1.31×10^5^	0.9997	0.30	1.00	101.66	2.66
Raffinose	0.5-10	*Y*=6.81×10^5^ *X*+3.97×10^4^	0.9999	0.09	0.30	100.45	3.36
Maltotriose	10-115	*Y*=4.31×10^5^ *X*+3.08×10^6^	0.9991	0.09	0.30	101.25	1.84
Maltotetraose	8-80	*Y*=1.29×10^5^ *X*-4.01×10^4^	0.9997	0.30	1.00	100.98	1.87
Stachyose	2-18	*Y*=9.83×10^5^ *X*+3.43×10^5^	0.9997	0.12	0.40	100.16	1.74

*Y*： peak area； *X*： mass concentration， mg/L.

烟叶样品基质复杂、干扰大，为进一步验证该方法测定烟叶样品中26种可溶性糖的可行性，选择No. 3、No. 18和No. 42号样品进行低、中、高3个水平的加标回收试验，每个样品平行3次。结果表明，在3个加标水平下，26种可溶性糖的加标回收率为92.39%~111.75%，相对标准偏差为0.87%~4.65%，表明该方法具有优异的准确度和精密度，可用于烟叶中可溶性糖的含量测定。

### 2.3 烟叶样品中可溶性糖类定量及差异分析

#### 2.3.1 定量分析

结合自建数据库，对不同产地、等级及年份的59种烟叶样品中可溶性糖种类进行定性筛查，并对所筛查出的可溶性糖进行定量。图6为3号和18号样品中目标物的提取离子流图，两个样品中所有被检测到的目标物色谱峰均实现了完全分离且峰形对称。如表3所示，共检测出6种可溶性糖，分别为葡萄糖、果糖、蔗糖、肌醇、山梨糖醇、赤藓糖醇，与高效液相色谱法^［19］^对3号和18号样品测定所得葡萄糖、果糖、蔗糖含量基本一致。各可溶性糖在不同产地、等级和年份间存在显著差异，其中果糖和葡萄糖在所有烟叶样品中含量均较高，蔗糖和肌醇次之，山梨糖醇和赤藓糖醇含量最低；从产地分布分析，黑龙江地区烟叶可溶性糖含量普遍较高，如56和57号样品所检测出的可溶性糖含量分别为284.971 mg/g和295.601 mg/g，而贵州、湖南等部分地区的可溶性糖含量相对较低，如42号样品（185.165 mg/g）、50号样品（160.741 mg/g）；年份方面，2021年和2022年样本可溶性糖含量整体高于2020年。此外，即使同一产地、等级和年份的不同样本间，可溶性糖含量也存在一定波动，推测与烟叶的生长环境、成熟度、调制和陈化等因素有关。

**图6:**
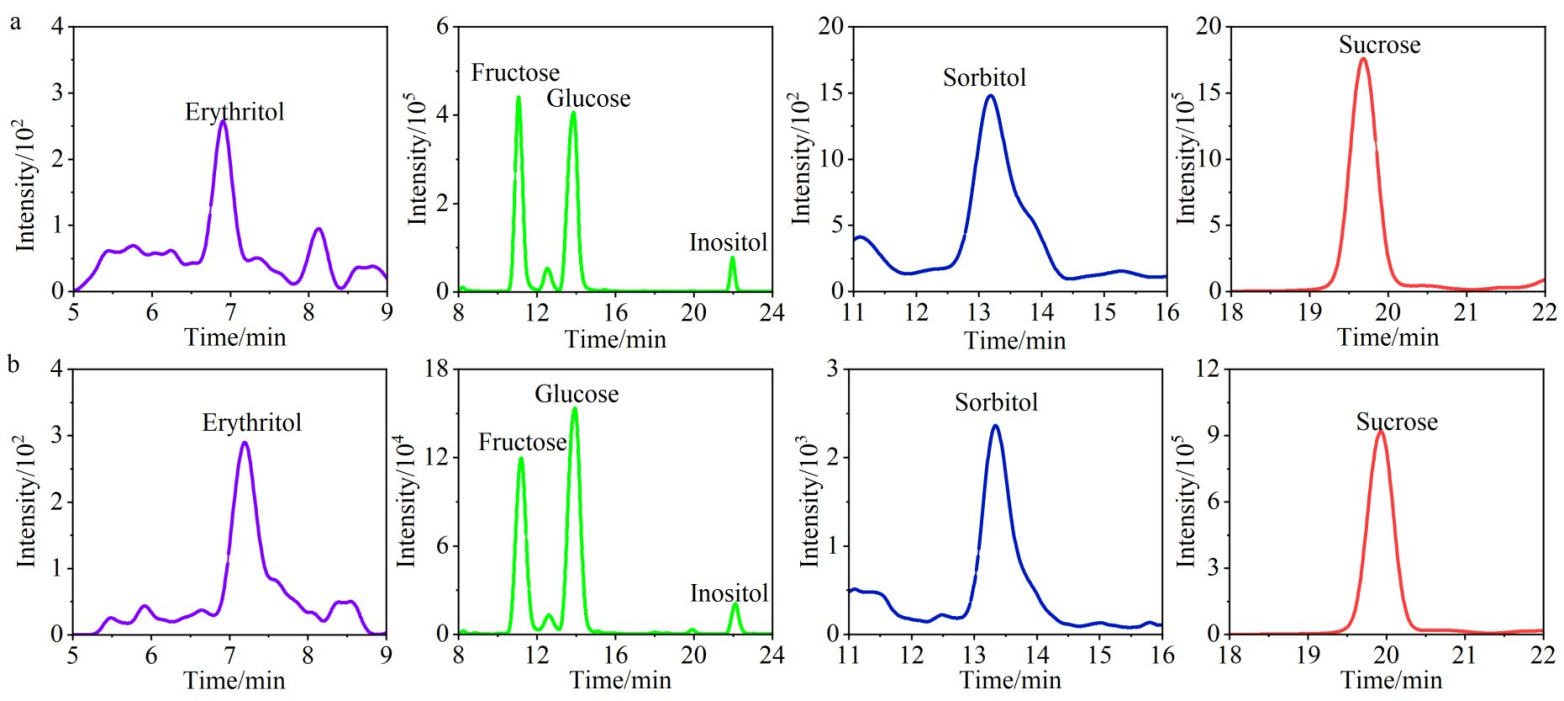
（a）3号和（b）18号烟叶样品的提取离子流色谱图（*n*=3）

**表 3:** 59种烟叶样品中可溶性糖的含量（*n*=5）

No.	Origin	Grade	Year	Contents/（mg/g） （RSDs/%）						Total content/ （mg/g）
				Glucose	Fructose	Sucrose	Inositol	Sorbitol	Erythritol	
1	America	#7 FCB3	2020	75.20 （3.21）	94.45 （2.25）	3.734 （4.23）	13.09 （0.51）	0.610 （4.55）	0.090 （4.38）	187.174
2	Brazil	#51 MBOA-GD	2020	75.97 （0.64）	102.6 （0.94）	3.425 （2.31）	12.37 （0.39）	0.500 （2.47）	0.117 （3.35）	194.982
3	Zimbabwe	#70 L1O/C	2020	73.58 （1.70）	106.6 （0.71）	5.580 （2.74）	7.923 （0.54）	0.981 （3.20）	0.163 （2.46）	194.827
4	Zimbabwe	#74 L1O/C	2020	76.64 （1.34）	107.1 （0.60）	4.625 （6.87）	7.271 （0.13）	1.053 （4.17）	0.183 （1.89）	196.872
5	Zimbabwe	#76 L1O	2020	72.72 （0.04）	106.4 （0.47）	7.440 （3.44）	8.194 （0.14）	1.152 （3.75）	0.188 （2.97）	196.094
6	Zimbabwe	#139 L1LA	2021	75.52 （0.97）	91.30 （0.89）	5.370 （5.47）	9.033 （0.02）	1.282 （2.07）	0.232 （4.57）	182.737
7	Zimbabwe	#140 L1OA	2021	82.17 （1.75）	104.4 （0.55）	9.457 （0.31）	15.56 （0.17）	0.453 （3.70）	0.112 （2.01）	212.152
8	Yunnan	C1F （A）	2021	82.06 （1.45）	129.2 （0.10）	13.27 （1.37）	10.08 （0.04）	0.124 （3.57）	0.035 （4.38）	234.769
9	Yunnan	C2F （A）	2021	90.78 （0.11）	109.6 （0.39）	17.34 （0.18）	9.337 （0.10）	0.137 （1.13）	0.025 （4.31）	227.219
10	Yunnan	C3F （A）	2021	96.46 （1.00）	131.0 （1.27）	13.64 （3.54）	13.96 （0.37）	0.111 （2.48）	0.024 （3.74）	255.195
11	Yunnan	C4F	2021	64.84 （2.80）	99.72 （0.62）	6.348 （3.38）	9.360 （0.25）	0.119 （4.21）	0.022 （3.44）	180.409
12	Yunnan	B1F （A）	2022	81.10 （0.84）	109.7 （0.52）	16.59 （0.92）	10.27 （3.89）	0.425 （4.15）	0.054 （3.11）	218.139
13	Yunnan	C1F （JD）	2020	77.19 （1.28）	129.1 （0.62）	3.335 （4.31）	10.69 （3.18）	0.581 （3.19）	0.238 （1.57）	221.134
14	Yunnan	C2F	2020	72.01 （1.06）	112.0 （0.53）	2.640 （3.95）	10.51 （3.80）	0.597 （4.15）	0.169 （3.96）	197.926
15	Yunnan	C2F （A）	2021	81.56 （0.88）	111.6 （0.75）	24.52 （0.48）	19.51 （1.75）	0.372 （2.22）	0.055 （3.61）	237.617
16	Yunnan	C3F （C2JD）	2021	90.91 （0.53）	115.5 （0.44）	16.54 （3.28）	16.55 （1.74）	0.195 （3.86）	0.037 （3.94）	239.732
17	Yunnan	B1F （B1）	2021	75.21 （0.41）	112.1 （0.43）	8.419 （0.12）	8.354 （0.28）	0.427 （3.03）	0.076 （4.24）	204.586
18	Yunnan	C3F （B）	2021	93.15 （0.02）	114.1 （0.51）	8.199 （1.65）	10.56 （1.09）	0.499 （0.51）	0.124 （1.08）	226.632
19	Yunnan	X2F	2021	61.23 （0.42）	93.72 （0.14）	5.270 （1.88）	10.23 （0.39）	0.335 （3.32）	0.009 （2.13）	170.794
20	Yunnan	C3L	2022	70.80 （1.17）	93.13 （0.46）	14.24 （4.34）	7.407 （1.07）	0.452 （4.03）	0.005 （3.32）	186.034
21	Yunnan	C1F （A）	2020	111.4 （0.85）	111.5 （0.69）	14.69 （1.64）	7.716 （2.63）	0.506 （4.14）	0.004 （2.59）	245.816
22	Yunnan	B2F （B1）	2022	59.24 （0.72）	81.42 （0.63）	8.460 （3.07）	6.313 （2.69）	0.512 （1.83）	0.037 （2.64）	155.982
23	Yunnan	C3F （C1）	2022	99.87 （0.95）	104.0 （0.93）	22.57 （1.57）	11.82 （3.22）	0.441 （1.16）	0.006 （4.34）	238.707
24	Yunnan	C2F	2022	89.66 （1.39）	94.69 （0.66）	23.77 （3.24）	8.442 （2.55）	0.358 （3.00）	0.002 （2.37）	216.922
25	Yunnan	C3F （C1）	2020	87.40 （0.70）	95.04 （0.34）	11.49 （4.57）	7.574 （1.11）	0.464 （4.02）	-	201.968
26	Yunnan	B2F （C）	2021	55.02 （0.53）	70.82 （0.62）	15.20 （4.48）	4.477 （0.93）	0.414 （4.38）	-	145.931
27	Yunnan	C4F	2022	66.09 （1.32）	81.09 （0.23）	15.41 （3.25）	6.482 （3.13）	0.374 （4.10）	-	169.446
28	Yunnan	C2F （G）	2019	76.15 （0.78）	95.20 （0.44）	5.250 （2.28）	7.708 （1.34）	0.748 （4.52）	0.093 （1.20）	185.149
29	Yunnan	C2F （A）	2022	87.63 （0.86）	96.32 （0.41）	11.17 （3.10）	8.377 （0.62）	0.454 （4.39）	0.083 （3.99）	204.034
30	Yunnan	B1F （A）	2022	67.27 （1.00）	80.13 （0.92）	7.515 （2.48）	6.498 （1.11）	0.639 （2.44）	0.061 （3.49）	162.113
31	Yunnan	B1F （B2）	2022	60.84 （1.08）	75.09 （1.08）	5.130 （1.37）	4.321 （3.45）	0.535 （4.20）	0.058 （3.51）	145.974
32	Sichuan	C2F （A）	2021	94.59 （1.01）	121.2 （0.31）	15.48 （1.25）	8.160 （3.05）	0.653 （4.24）	0.061 （2.42）	240.144
33	Sichuan	C2F （A）	2019	61.47 （1.37）	127.4 （0.41）	8.240 （2.27）	3.590 （1.18）	0.619 （2.66）	0.116 （3.33）	201.435
34	Sichuan	C2F （AJD）	2021	66.15 （1.24）	98.98 （0.86）	15.36 （2.46）	6.745 （3.44）	0.416 （2.49）	0.044 （4.10）	187.695
35	Sichuan	C2F （T）	2022	85.29 （1.26）	113.7 （0.99）	17.38 （1.38）	7.948 （2.97）	0.543 （4.39）	0.137 （0.52）	224.998
36	Sichuan	C3F （A）	2021	109.5 （1.18）	137.2 （0.82）	14.68 （2.20）	8.047 （2.96）	0.508 （2.31）	0.099 （3.56）	270.034
37	Sichuan	C3F （AJD）	2021	105.3 （0.58）	117.7 （0.67）	21.38 （1.32）	8.380 （3.94）	0.563 （4.06）	0.046 （3.41）	253.369
38	Sichuan	C3F （A）	2022	93.72 （1.16）	106.4 （0.50）	21.70 （3.03）	8.330 （2.32）	0.429 （4.06）	0.046 （3.90）	230.625
39	Sichuan	C3F （C2）	2021	91.70 （0.85）	124.1 （2.07）	13.52 （1.78）	10.79 （4.32）	0.121 （3.99）	0.029 （2.53）	240.260
40	Guangdong	C4F （C1）	2022	112.7 （0.62）	120.7 （0.70）	6.443 （4.30）	15.50 （2.44）	0.542 （3.11）	0.167 （4.26）	256.052
41	Guangdong	C3F （C1）	2022	101.3 （0.83）	110.1 （0.67）	6.520 （3.80）	13.50 （3.48）	0.595 （4.39）	0.152 （0.85）	232.167
42	Guizhou	C3F	2022	77.80 （1.53）	97.20 （1.03）	6.021 （1.73）	4.144 （4.59）	-	-	185.165
43	Guizhou	C3F （A）	2021	80.66 （1.39）	87.31 （1.38）	6.258 （1.00）	9.408 （0.14）	0.667 （4.36）	0.023 （3.80）	184.326
44	Guizhou	C3F （C1）	2022	75.27 （0.96）	89.37 （2.29）	6.239 （3.59）	8.343 （3.76）	0.607 （3.69）	0.027 （3.87）	179.856
45	Guizhou	C2F （G）	2021	97.27 （1.09）	103.4 （1.01）	16.34 （0.52）	7.342 （0.59）	0.546 （3.61）	0.045 （4.03）	224.943
46	Fujian	C2F （A）	2021	115.3 （1.11）	121.4 （0.56）	12.81 （0.83）	7.391 （1.17）	0.265 （3.07）	-	257.166
47	Fujian	C3F （C1）	2022	116.5 （0.43）	122.8 （0.30）	6.351 （2.54）	9.294 （1.26）	0.371 （1.22）	-	255.316
48	Fujian	C2F （A）	2022	115.5 （0.79）	124.9 （0.31）	13.78 （0.59）	13.16 （2.88）	0.154 （3.00）	-	267.494
49	Fujian	C3F （A）	2021	118.7 （0.30）	123.0 （1.33）	5.933 （2.23）	12.51 （0.55）	0.261 （2.17）	-	260.404
50	Hunan	X2F	2020	65.79 （1.08）	84.06 （1.23）	1.050 （4.35）	9.250 （3.21）	0.555 （3.60）	0.036 （3.17）	160.741
51	Hunan	C3F （C1）	2020	75.16 （1.97）	99.06 （1.63）	2.323 （4.19）	6.851 （2.61）	0.511 （4.52）	0.051 （3.75）	183.956
52	Hunan	B1F （A）	2020	57.72 （1.34）	81.77 （1.26）	0.259 （3.76）	8.522 （2.93）	0.617 （3.00）	0.055 （4.50）	148.943
53	Hunan	C3F （C1）	2021	71.70 （1.71）	97.36 （1.08）	2.463 （3.89）	6.990 （2.50）	0.629 （2.54）	0.037 （3.31）	179.179
54	Hunan	C3F	2021	96.79 （0.34）	139.9 （0.24）	4.373 （3.79）	11.60 （1.76）	-	-	252.663
55	Hunan	C3F	2021	85.48 （1.18）	100.6 （1.14）	3.470 （4.62）	6.503 （0.94）	0.523 （2.04）	0.085 （1.80）	196.661
56	Heilong-jiang	C2F	2022	129.9 （0.39）	127.4 （0.59）	17.56 （2.67）	9.274 （1.28）	0.653 （2.44）	0.184 （2.00）	284.971
57	Heilong-jiang	C3F	2021	133.5 （0.64）	128.2 （0.87）	23.74 （2.62）	9.387 （3.89）	0.604 （3.44）	0.170 （3.95）	295.601
58	Shaanxi	C3F	2021	84.55 （1.25）	90.70 （2.29）	37.50 （2.27）	5.215 （1.55）	-	-	217.965
59	Shandong	C3F	2021	78.29 （0.65）	106.5 （0.87）	4.461 （3.32）	4.284 （1.98）	-	-	193.535

-： <LOQ.

#### 2.3.2 不同部位烟叶中可溶性糖成分差异分析

为探究不同部位烟叶中可溶性糖成分差异，选取同一年份、同一产地、不同部位的云南普洱烟叶样品进行考察（上部：No.17、中部：No.15、下部：No.19），每组设置5个平行样本。将筛查出的可溶性糖类成分及其含量进行PCA和HCA。PCA分析结果证明该模型有优异的拟合度与预测能力（图7a，*R* ^2^ *X*=0.996，*Q* ^2^=0.949）。为进一步验证PCA分析结果，对所选烟叶样品进行HCA分析，结果如图7b所示，与PCA分析结果一致，15个样品明显聚为3类，与烟叶的3个部位完全对应，表明不同部位烟叶中可溶性糖含量存在显著差异。

**图7:**
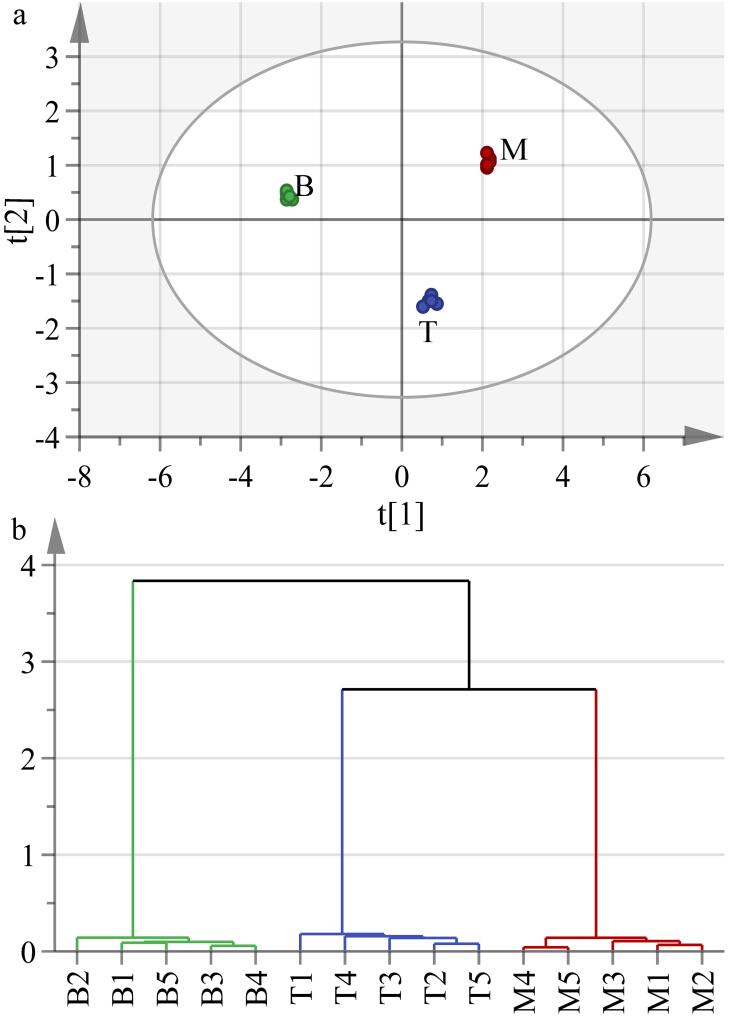
不同部位烟叶中可溶性糖类成分的（a）主成分分析图和（b）聚类分析图（*n*=5）

### 2.4 方法性能比较

本研究建立的方法可在无标准品条件下实现26种可溶性糖类化合物的快速、高通量定性与定量分析。与文献报道和国家现行食品及烟草中可溶性糖标准检测方法相比^［7，16，17，19］^，建立的方法在灵敏度、检测覆盖范围和定量准确性方面均具有明显优势，适用于复杂样品中可溶性糖的高通量快速筛查与准确定量（表4）。

**表 4:** 本方法与其他方法对比

Detection method	Sample	Number of detected sugars	LOQ/（mg/L）	Ref.
HPLC-ELSD	food	5	500	［^19^］
Ion chromatography	food	5	10	［^19^］
Continuous flow method	tobacco	total content	/	［^7^］
HPLC-ELSD	tobacco	4	4.1-7.1	［^16^］
HPLC-MS/MS	tobacco	7	0.02-0.05^*^	［^17^］
UPLC-Q-TOF-MS	tobacco	26	0.03-20	this study

ELSD： evaporative light scattering detector； /： cannot be quantified； * LOD.

## 3 结论

本研究建立了一种定性定量检测烟叶样品中26 种可溶性糖成分和含量的方法。该方法整合了UPLC-Q-TOF-MS的高通量、高灵敏度优势与自建数据库的高效定性能力，具有准确度和精确度高、定量限低等优点。用该方法成功获得不同来源烟叶样品中可溶性糖的成分和含量。统计学分析证明不同部位的可溶性糖含量存在显著差异，凸显了可溶性糖在烟草分类与品质判别中的重要性。该方法无需标准品即可实现烟叶中可溶性糖高通量快速筛查和准确定量，有效克服了传统可溶性糖测定技术分析效率低、标准品依赖性强等局限性，为烟叶中可溶性糖的成分和含量测定提供一种高效、可靠且便捷的方法。然而，由于烟叶样品基质复杂、部分可溶性糖含量低，在实际烟叶样品中仅测到6种可溶性糖。后续可通过优化样品前处理方式如富集、浓缩，以进一步提高方法灵敏度并降低基质干扰，为实际烟叶样品中低含量糖提供更可靠的方法支持。
